# Update: Microdialysis for Monitoring Cerebral Metabolic Dysfunction after Subarachnoid Hemorrhage

**DOI:** 10.3390/jcm10010100

**Published:** 2020-12-30

**Authors:** Pierce Spencer, Yinghua Jiang, Ning Liu, Jinrui Han, Yadan Li, Samuel Vodovoz, Aaron S. Dumont, Xiaoying Wang

**Affiliations:** Clinical Neuroscience Research Center, Department of Neurosurgery, Tulane University School of Medicine, New Orleans, LA 70112, USA; pspencer1@tulane.edu (P.S.); nliu3@tulane.edu (N.L.); jhan10@tulane.edu (J.H.); yli72@tulane.edu (Y.L.); svodovoz@tulane.edu (S.V.); adumont2@tulane.edu (A.S.D.)

**Keywords:** subarachnoid hemorrhage, cerebral microdialysis, brain metabolism, brain bioenergetics, metabolic dysfunction

## Abstract

Cerebral metabolic dysfunction has been shown to extensively mediate the pathophysiology of brain injury after subarachnoid hemorrhage (SAH). The characterization of the alterations of metabolites in the brain can help elucidate pathophysiological changes occurring throughout SAH and the relationship between secondary brain injury and cerebral energy dysfunction after SAH. Cerebral microdialysis (CMD) is a tool that can measure concentrations of multiple bioenergetics metabolites in brain interstitial fluid. This review aims to provide an update on the implication of CMD on the measurement of metabolic dysfunction in the brain after SAH. A literature review was conducted through a general PubMed search with the terms “Subarachnoid Hemorrhage AND Microdialysis” as well as a more targeted search using MeSh with the search terms “Subarachnoid hemorrhage AND Microdialysis AND Metabolism.” Both experimental and clinical papers were reviewed. CMD is a suitable tool that has been used for monitoring cerebral metabolic changes in various types of brain injury. Clinically, CMD data have shown the dramatic changes in cerebral metabolism after SAH, including glucose depletion, enhanced glycolysis, and suppressed oxidative phosphorylation. Experimental studies using CMD have demonstrated a similar pattern of cerebral metabolic dysfunction after SAH. The combination of CMD and other monitoring tools has also shown value in further dissecting and distinguishing alterations in different metabolic pathways after brain injury. Despite the lack of a standard procedure as well as the presence of limitations regarding CMD application and data interpretation for both clinical and experimental studies, emerging investigations have suggested that CMD is an effective way to monitor the changes of cerebral metabolic dysfunction after SAH in real-time, and alternatively, the combination of CMD and other monitoring tools might be able to further understand the relationship between cerebral metabolic dysfunction and brain injury after SAH, determine the severity of brain injury and predict the pathological progression and outcomes after SAH. More translational preclinical investigations and clinical validation may help to optimize CMD as a powerful tool in critical care and personalized medicine for patients with SAH.

## 1. Introduction

Subarachnoid hemorrhage (SAH) is an acute cerebrovascular disease. Despite accounting for only 5% of all stroke cases [1,2], some features render SAH as one of the most devastating diseases. Not only does SAH tend to affect individuals of a younger age compared to other types of stroke, but it also causes high mortality and significant morbidity [3,4]. Approximately 12% of patients die prior to receiving medical management, and 50% within 30 days of SAH. Half of the survivors suffer from a permanent disability [5]. Etiologically, approximately 85% of cases are caused by the rupture of a cerebral aneurysm [6]. Followed by the sudden rupture, blood accumulates within the subarachnoid space, resulting in a series of pathophysiological changes, impacting both brain vasculature and parenchyma, causing secondary brain injury [7]. Mechanisms mediating secondary brain injury after SAH are multifactorial. Initially, delayed vasospasm was considered to be a primary factor resulting in the deterioration of neurological outcomes in SAH patients. However, successful relief of vasospasm has failed to improve functional outcomes in clinical trials [8]. Later, the concept called early brain injury (EBI), which is defined as pathophysiological changes within the first 72 h after the onset of bleeding, has been emphasized and gained increasing attention by the SAH research community [5]. Importantly, dysregulation of energy metabolism in brain tissue is one of the driving forces contributing to pathological changes in various acute brain injuries, such as traumatic brain injury [9]. Similarly, cerebral energy dysfunction occurs rapidly and could extend to a prolonged period of time after SAH [10]. Hence, measuring and monitoring the alterations in post-SAH energy metabolism inside the brain would be essential for not only improving patient management in clinical practice but for understanding the mechanisms underlying the post-SAH cerebral energy dysregulation, as well as its correlation with outcomes [11].

Cerebral microdialysis (CMD) is an approach using a concentric probe with a semipermeable membrane to collect brain interstitial fluid (ISF) that has been extensively used in both clinical and experimental studies for analyzing the status of cerebral energy metabolism [10]. Solutes, such as energy metabolites, neurotransmitters, or amino acids in ISF diffuse down the concentration gradient moving across the semipermeable membrane and are eventually collected as dialysate or analyte, followed by the concentration measurement by using analytical chemistry techniques, such as high-performance liquid chromatography with mass spectrometry. Indeed, using CMD allows researchers to see a more comprehensive picture of bioenergetic metabolism during and after SAH and help clinicians guide patient treatment and monitor brain metabolic changes preceding the clinical deterioration [12]. Simultaneously, other monitoring data, such as intracranial pressure (ICP), cerebral perfusion pressure, and brain tissue oxygen tension, are collected together with CMD data and may thus provide a direct way to measure the process of energy failure at the cellular level [13]. Interestingly, several studies have also demonstrated that monitoring patients with brain injury by CMD might be beneficial for reducing the mortality rate [14,15]. This review aims to discuss the current advances and the potential for utilizing CMD in both clinical and experimental studies. From a translational perspective, we believe that CMD is an important approach for further investigating the SAH-induced cerebral energy dysfunction, and eventually, finding an effective intervention for treating SAH.

## 2. Cerebral Metabolic Dysfunction after SAH

As mentioned above, the current concept dividing the post-SAH pathophysiological process into two phases: the phase of EBI occurred within the first 72 h after the onset of ictus, followed by a delayed phase typically occurs 3–14 days after SAH where the cerebral vasospasm and subsequent delayed cerebral ischemia (DCI) ensue and affect up to half of the SAH patients [16]. Both phases involve prominent derangements in the brain’s bioenergetic profile [17] that can be measured intracellularly and extracellularly.

### 2.1. Cerebral Metabolic Dysfunction in Early Phase after SAH

The definition of EBI includes the evaluation of initial clinical symptoms, neuroimaging findings, and cerebral electrophysiological and/or metabolic changes using multimodal neuromonitoring, including CMD. During the initial vessel rupture, blood enters the subarachnoid space, increasing ICP, leading to a reduction in cerebral perfusion pressure and cerebral blood flow (CBF) [18]. This clinical pattern has been successfully replicated in rodent studies, implying that the EBI’s pathophysiology between the two species share similar mechanisms [18,19]. The sudden changes in CBF and cerebral perfusion deficit result in multiple metabolic disturbances [18]. In fact, some studies have demonstrated the similarities between SAH and transient global cerebral ischemia [20]. Normal functions of neurons heavily rely on the oxidative phosphorylation within the mitochondria via the electron transport chain for ATP generation. Acute cerebral ischemia depletes oxygen supply and reduces mitochondrial respiration, forcing the shift from oxidative phosphorylation to enhanced anaerobic glycolysis in neurons and other brain-resident cells, followed by an accumulation of lactate developing a local or diffuse acidosis [21]. Additionally, dramatic reductions in intracellular adenosine triphosphate (ATP) derived from the energy failure after SAH leads to ion channel dysfunction and disruption of the normal cell membrane potential. All these processes could enhance the production of reactive oxygen species (ROS) and brain tissue damage [22]. Moreover, cerebral hypoperfusion has been correlated with an increase in extracellular glutamate [23,24]. Excess extracellular glutamate that cannot be recycled causes the prolonged activation of both NMDA and AMPA receptors. The following supraphysiological influx of calcium into neurons and glial cells can eventually lead to apoptosis [25]. Astrocytic apoptosis, accompanied by the loss of astrocytic foot processes, plays a key role in BBB permeability after SAH [26]. CMD data obtained during the acute phase of SAH has shown a sharp reduction in glucose, while there was an elevation in glutamate, lactate, and lactate to the pyruvate ratio (LPR), which is a marker indicating the cerebral ischemia-related energy crisis [27]. 

In addition, non-ischemic mechanisms also mediate cerebral energy dysfunction in the EBI phase after SAH since clinical data, based on microdialysis analysis, showed that more pronounced CMD abnormalities, including the increased LPR, were observed in SAH patients with hyperemic or normal CBF. Extravascular hemolysis of red blood cells in subarachnoid space causes free hemoglobin to enter the cerebral interstitial system [22]. Consequently, the hemoglobin scavenges nitric oxide, reducing its availability to endothelium and smooth muscle cells [22] and disrupting the ionic homeostasis of smooth muscle cells, resulting in vasoconstriction of the cerebrovasculature and further energy insufficiency [28]. Hence, multiple factors contribute to the cerebral energy dysregulation involved in EBI after SAH. However, further investigations targeting the interaction and association between these factors are still needed.

### 2.2. Cerebral Metabolic Dysfunction in Late Phase after SAH

DCI has the highest incidence 3–14 days after ictus [29]. Previously, cerebral vasospasm has been considered a culprit in DCI. However, many clinical trials have failed to improve post-SAH outcomes by using anti-vasospasm agents [30]. Recent evidence has shown a weak correlation between the vasospasm and the location of DCI [31]. The current concept deems DCI a multifactorial process, including microvasculature dysfunction, microthrombi formation, inflammation, and cortical spreading depolarizations (SDs) [31,32]. Previous data demonstrated that persistent constriction of brain arterioles could last up to 72 h post-SAH [33]. Further evidence showed that the development of significant microthrombi in these constricted brain arterioles compared to arterioles without constriction [33], suggesting the cerebrovascular dysfunction starting at the early phase after SAH may sustain to an extended period of time and also strengthening the correlation between EBI components and the development of DCI [34]. Additionally, the inflammatory reaction induced by the interaction of hemoglobin released from lysed red blood cells and surrounding brain tissue can elicit the infiltration of peripheral immune cells and subsequent production of pro-inflammatory cytokines into the adjacent brain parenchyma [35]. 

During the late phase of subarachnoid hemorrhage, metabolic profiling using microdialysis has shown a similar pattern as the early phase after SAH [27]. Interestingly, in addition to the reduction in glucose and elevation of lactate, glutamate, and glycerol, an aggravation of energy crisis is reflected by a further increase in LPR, which can be detected during this delayed phase after SAH and may be partially due to the decreased level of pyruvate, suggesting the occurrence of delayed cerebral ischemia. Moreover, LPR deterioration can be detected as early as 16 h before DCI, rendering the possibility for early prevention of severe complications in the delayed phase of SAH [27].

## 3. CMD in Monitoring Cerebral Metabolic Dysfunction after SAH

Clinically, CMD can be used during the entire perioperative period in SAH patients, particularly for patients with poor-grade SAH [36]. While CMD is not currently an essential component of standard post-SAH care, CMD readings provide a wide array of useful information regarding bioenergetic metabolism inside the post-SAH brain. For instance, the most recent consensus from the 2014 international microdialysis forum concludes a set of average metabolite concentrations and treatment recommendations based on CMD monitoring for various acute brain injuries, including SAH [13]. 

### 3.1. Key Metabolic Parameters Measured by CMD

By collecting brain ISF samples, several important metabolites related to brain function and metabolism can be analyzed by CMD. Under normal conditions, glucose is the primary energy source for the brain, and neurons use a large portion of glucose for oxidative phosphorylation. However, under pathological conditions, such as in the early phase after SAH, anaerobic glycolysis starts to predominate in brain residual cells, called hyperglycolysis, as the discontinuation of oxygen and glucose supply caused by reduced CBF [37,38], which is reflected by the marked decrease in the concentration of glucose in brain interstitial fluid [39]. Lactate is the major product of glycolysis and is linked to the post-SAH outcome. Some clinical studies showed that a metabolic profile coinciding with hyperglycolysis, defined as ISF concentrations of lactate >4 mmol/L and pyruvate >119 μmol/L with brain tissue oxygen pressure >20 mm Hg, is correlated with better clinical outcomes in SAH patients [40,41]. However, the elevation of brain lactate level, is categorized into two patterns: nonhypoxic hyperglycolysis, which is associated with better outcomes, or hypoxic hyperglycolysis related to the poor outcome in SAH patients. The latter pattern is associated with more severe ischemia and decoupling of glycolysis and oxidative phosphorylation that can be indicated by significantly increased LPR [21]. 

Glutamate is a major excitatory neurotransmitter that plays vital roles in normal brain functions. However, excessive extracellular glutamate can cause excitotoxicity triggering neuronal death. Under physiologic conditions, astrocytes are responsible for the reuptake of excess glutamate released from neurons through an energy-dependent process [42]. Thus, glutamate concentration in extracellular space is used as a marker to determine the status of energy metabolism in the brain, since abnormal increases in extracellular glutamate may reflect the energy failure and disruption of the glutamine-glutamate cycle between neurons and astrocytes [43]. Additionally, glycerol is a product of cell membrane degeneration, and its level in ISF has been considered an indicator of membrane cell damage after SAH. Higher levels of glycerol were reported in patients with poor-grade SAH [44]. 

### 3.2. Clinical Application of CMD in SAH

As mentioned above, CDM is commonly used in the neurointensive care unit for multiple types of brain injury. However, CMD monitoring is typically only used in severe SAH cases because it is an invasive procedure and not a current standard component for SAH management [45]. Cerebral microdialysis measures extracellular fluid concentrations of the lactate to pyruvate ratio, glutamate, glucose, and glycerol. Monitoring cerebral metabolism can help experienced clinicians to make treatment decisions or monitor potential neurological deterioration. CMD can measure not only ISF concentrations of various metabolites from energy metabolism in the brain, but also analyze other factors such as specific inflammatory cytokines and matrix metalloproteinase-9 (please refer to Table 1 for all the significant clinical findings based on CMD monitoring within the past two years.) [46,47]. Thus, continued research into potential biochemical markers of EBI and DCI after SAH based on CMD may provide a promising way for better understanding the involvement of energy dysregulation in post-SAH pathophysiology.

There has been contradictory evidence in the literature regarding whether CMD can detect cerebral energy metabolism disturbances preceding EBI, but it has shown promising results in studies focusing on DCI [49,50,55]. Metabolite concentrations during the acute phase after SAH show decreased glucose while glutamate, lactate, and glycerol increase [58]. Interestingly, DCI can be predicted with an 88% specificity and 94% sensitivity if the probe around the ruptured vessel detects the LPR elevation, lactate to glycerol ratio greater than 20%, and a subsequent 20% increase in glycerol within one day [59]. Conversely, the LPR and glutamate levels may fluctuate during the phase of EBI [46]. Excitingly, a clinical study recruiting 97 SAH patients, has found that CMD is a safe technique for monitoring regional cerebral ischemia. The changes of metabolites indicative of cerebral ischemia, including the low glucose level combined with significant elevations in lactate, LPR, glutamate, and glycerol, preceding the onset of symptomatic vasospasm were detected in the majority of SAH patients with delayed ischemic neurologic deficits, which are in good agreement with the clinical course of these patients [60]. Later, the author replicated the consistent result in another clinical studies, including 44 SAH patients [24]. In the consensus statement mentioned above regarding the indication of CMD in SAH, the author stated that CMD could be used as a primary monitoring device in patients with poor-grade SAH that also allows clinicians to detect secondary neurological deterioration [13]. Meanwhile, the statement proposed that the most useful markers of brain metabolism after SAH include ISF concentration of glucose, glutamate and glycerol, and the LPR. While the use of CMD has shown to improve functional outcomes, including the reduced mortality rate and better Glasgow coma scales 3–6 months post-injury, in patients with traumatic brain injury [15], the relationship between the CMD monitoring and clinical outcomes in SAH patients remain to be elucidated. Also, it has been reported that CMD may help to assess the efficacy of SAH-related interventions [54,57]. Furthermore, despite a recent article proposing the standard CMD reporting recommendations [61], a more comprehensive meta-analysis might be required for further standardizing the reporting of CMD results from both clinical and experimental studies due to the current variability in reported data of CMD in the literature. Indeed, more research and review articles are essential to allow us to build a more robust consensus for the standard use of CMD in patients with SAH or other types of brain injury and integrating CMD into the standard of SAH management.

### 3.3. Association between CMD Readouts and Post-SAH Pathophysiology and Outcome

Despite decades of CMD application in the management of SAH patients in the neurosurgery department, the correlation between CMD readings and post-SAH pathophysiology remains to be further elucidated. Indeed, previous clinical studies have demonstrated the ability of CMD readings to predict or monitor early pathophysiological events after SAH. For instance, the rapid occurrence of global cerebral edema after the ictus, which has been considered an independent risk factor for mortality and poor outcome after SAH, is accompanied by a series of cerebral metabolic changes, including the significantly higher LPR and lower cerebral glucose level [12]. Among those SAH patients with acute focal neurological deficits, which is partially the result of cerebral edema, CMD also detected the increased lactate, LPR, glutamate and glycerol [24], suggesting that the cerebral metabolic dysregulation persists and contributes to multiple early pathophysiological events after SAH. Additionally, the cortical SDs, as an important early electrophysiological change after SAH, has also been associated with brain energy failure and delayed cerebral ischemia after SAH [62]. A corresponding reduction in glucose level and elevation in lactate level in the brain tissue has been demonstrated during the phase of SDs [63]. In general, the higher LPR is a common feature frequently observed in SAH patients with poor clinical grades or worse functional outcomes [27,52]. For a more detailed and systematic review regarding correlations between CMD readouts and post-SAH outcomes, please refer to other well-written articles [27,58].

Another aspect of post-SAH pathophysiology that is worth mentioning is the neuroinflammation, since it also closely associates with the state of brain energy metabolism, while the immunometabolism in the brain has also been proven to play a pivotal role in various types of brain injury [64]. In addition to measuring energy metabolites, CMD is also capable of analyzing inflammation-related factors. The tumor necrosis factor (TNF)-α is a classic proinflammatory cytokine that plays a central role in neuroinflammation after brain injury. By using CMD, the relationship between the elevated extracellular level of TNF-α and the incidence of intraventricular hemorrhage after SAH, as well as the larger aneurysm size, has been established [65]. Further, the TNF-α level in extracellular space also correlates with the severity of delayed vasospasm after SAH [66]. Interleukin (IL)-6 is another important, predominantly proinflammatory cytokine in the context of SAH. A study recruited 38 consecutive SAH patients found that increased level of IL-6 in extracellular fluid reflects a pronounced local inflammatory response in the brain after SAH. Interestingly, the elevation of IL-6 accompanies the higher LPR and indicates the subsequent occurrence of DCI in symptomatic patients [67]. Another clinical study demonstrated that, in the presence of increased ICP, circulating IL-6 primarily originated from extracellular fluid in the brain after SAH [68]. In addition, a higher level of pro and mature form of matrix metalloproteinase-9 in extracellular space is related to the increased SAH severity and vasospasm [69]. Interestingly, high-grade neuroinflammation was also linked to the extracellular tau protein level, metabolic distress and delayed cerebral infarction [70]. Currently, both experimental and clinical studies using CMD to investigate the brain inflammation after SAH are limited but warranted.

### 3.4. Limitations and Future of Clinical Utilization of CMD

Limitations exist in the application of CMD in patients with neurological diseases. Since CMD is a relatively new modality of neuromonitoring, standards for its use in patients with severe neurological disorders have not been fully developed [71]. While previous studies have shown that CMD might be safe for monitoring the brain metabolism in neurocritical care unit with a relatively low risk of inducing intracerebral hemorrhage or infection by the CMD probe and catheter implementation [60,72], the invasive nature of CMD, including the craniotomy and probe implementation, still limits its wide use in SAH patients and requires specific training for physicians and nurses to perform this procedure in patients.

The location of the CMD probe is another important factor that needs to be well defined, while not only conducting the experiments, but also in analyzing the data. Technically, the CMD probe can only sample from a limited area of the brain tissue that is adjacent to it. The readings from CMD may not be simply expanded to the level of the entire cerebral metabolism. Current guidelines recommend CMD probe implementation in the frontal watershed of the hemisphere bearing the aneurysm or vascular territory that has the highest probability of developing secondary brain injury after SAH [13]. A recent clinical study found that the CMD probe located in the perilesional area, which is defined as the presence of abnormal signals within 1 cm of the tip to CMD probe based on the CT scan, is more likely to detect metabolic distress and mitochondrial dysfunction, whereas the CMD probe placed in normal-appearing brain parenchyma may generate more relevant metabolic information and provide higher prognostic value than perilesional area [48]. Interestingly, another study included 16 SAH patients demonstrated that contralateral hemisphere has a high rate of developing ischemic events compared to the ipsilateral hemisphere [51]. Hence, a more uniform recommendation for the CMD probe implantation that can reduce the heterogeneity of definitions and data interpretations in the literature is still needed. In addition, correlations between local CMD readouts in the brain and systemic changes after SAH also warrant a further investigation [53,56]. Indeed, the amount of analyte collected by the CMD probe only represents a fraction of the actual extracellular concentration inside the brain, since most flow rates do not allow for the complete diffusion of metabolites from the ISF into the dialysate [73]. At a commonly used flow rate for sample collection, e.g., 1μL/min, the ratio of the concentration of targeted molecules between the collected dialysate and the actual extracellular space, called the extraction fraction or relative recovery, is typically less than 40%, suggesting an inverse correlation between the flow rate and extraction fraction [74]. Certainly, by simply lowering the flow rate may allow for the more efficient exchange between dialysate and extracellular space and thus to increase the concentration of targeted molecules in collected dialysate. However, very slow flow rates are inappropriate for most studies due to not being able to collect a sufficient volume of the sample within a fixed period of time.

Several other factors also influence the extraction fraction, including the permeability and the probe membrane’s size. The permeability of the membrane is based on its porosity. Currently, the 20 kDa probe membrane is FDA approved for clinical use and it can typically measure energetic metabolites, as previously discussed. Further, the 100 kDa probe membrane enables the measurement of cytokines, matrix metalloproteinases, and other larger molecules in experimental studies. An increase in the permeability of the probe membrane results in a decrease in extraction fraction [75]. The probe size determines the surface area for diffusion. Accordingly, diffusion will occur much slower in a short and skinny probe than a long wide probe. Hence, it is crucial to understand how each of these variables affects the extraction fraction while interpreting the CMD data to avoid underestimation. Moreover, probe implementation can cause an inflammatory response by damaging the brain tissue surrounding it [76]. Accordingly, ISF samples collected within a short period of time after probe implementation may not accurately reflect the metabolic changes, only acute inflammation, and should be discarded.

The dialysis process can deplete target substances and other molecules in the area surrounding the CMD probe. This change in the local neurochemical environment may impact the basal levels and/or the pharmacological responsiveness of the substance under investigation [73]. For instance, some studies suggested that using neurotransmitter-free artificial cerebrospinal fluid for CMD perfusion may lead to an artificial concentration gradient of neurotransmitter and false detection of neurotransmitter changes in the probe area [77]. Hence, the technical issue in terms of avoiding false detection, while collecting an adequate amount of sample, is still a challenge in CMD application. In the clinic, dialysate is usually collected hourly, and advances have been made that allow for rapid analysis of dialysate every 30 s, thus reducing the sampling time and potential substance depletion [78]. Based on the concentration gradient-driving nature of the CMD, several other possibilities can be proposed. First, the administration of isotope-labeled energy metabolites while collecting the ISF samples through the CMD probe enables the researcher to quantify further the changes in different pathways of energy metabolism [79] and distinguish the different metabolic profiles in different types of brain cells after brain injury [80]. Second, CMD is superior in detecting the extracellular concentration of a drug in the brain after peripheral administration and is thus useful for determining the BBB permeability and pharmacokinetic parameters of a substance [81]. Third, localized pharmacotherapy through CMD is another possibility [82]. However, the value of this approach in SAH needs to be further validated.

### 3.5. Other Tools in Evaluating Cerebral Metabolic Dysfunction in SAH

While we mainly focused on CMD in this review, the other techniques used in clinical practice to monitor SAH patients include transcranial Doppler ultrasonography (TCD) and magnetic resonance imaging (MRI). A recent meta-analysis showed that TCD is highly sensitive for detecting vasospasm and subsequent DCI after SAH [83]. However, the sensitivity and specificity of TCD to detect vasospasm in arteries other than the middle cerebral artery are poor [84]. A recent meta-analysis concluded that MRI is useful in the diagnosis, but not in the prediction of DCI [85]. Excitingly, using MR spectroscopy along with MR perfusion imaging can evaluate the changes of brain metabolism and perfusion, respectively, in almost any region of the brain, which can be defined by the underlying anatomy or the relationship to the vascular territories. Combining such imaging modalities may allow us to further investigate the brain’s metabolic changes after SAH. A clinical study of 58 patients with SAH has detected, by using the aforementioned imaging combination, significant changes in neuronal mitochondrial injury, independent of perfusion deficits after SAH [86].

The combination of CMD with other monitoring techniques has been extensively tested in other brain injuries, such as traumatic brain injury. For instance, a clinical study that combined isotope-labeled CMD with nuclear magnetic resonance has successfully found that the post-trauma human brain can utilize lactate via TCA cycle and this combination also allowed the direct comparison of glycolysis and pentose phosphate pathway, another important energy metabolism pathway consuming glucose for anabolic biosynthesis and redox homeostasis instead of ATP production, after traumatic brain injury [79]. Another interesting experimental study using a similar combination approach has demonstrated a lactate storm following brain trauma may contribute to a rapid progression toward irreversible brain injury and pan-necrosis [87]. Unfortunately, such useful combination approaches have been scarcely investigated under SAH conditions.

Overall, each technique offers unique pros and cons. When used in conjunction with each other, clinicians can gain a bigger picture inside the metabolism disruption-related pathophysiology occurring after brain injury, particularly for SAH patients.

## 4. Preclinical Application of CMD in SAH Studies

Indeed, continuing attempts to advance our understanding of the SAH-related pathophysiology and pharmacological treatments through clinical studies alone is inefficient. Preclinical studies allow us to directly access brain tissue, which is not always feasible in clinical studies. Moreover, well designed and controlled experimental studies could significantly reduce the variability likely observed among SAH patients. Despite the importance of understanding the role of brain metabolism derangement after SAH, only a handful of preclinical studies have specifically focused on this scientific issue, particularly by using CMD. Thus far, all of these preclinical studies used rats for modeling SAH. It is probably due to the larger volume of the brain tissue and the feasibility of adequate ISF collection. Currently, there are two major methods for modeling SAH in animals. One is the endovascular filament-perforation, which closely simulates the process of vascular rupture and blood accumulation in subarachnoid space in human SAH. Another is the blood injection into subarachnoid space, which is more reproducible and controllable than the filament perforation model [88].

Using CMD combined with local cerebral blood flow and ICP monitor, Thomas et al. showed a marked ICP elevation with a sharp reduction of CBF and cerebral perfusion pressure, consistent with clinical observation. The author also investigated a post-SAH temporal profile of changes in various energy metabolites, including glutamate, lactate, and pyruvate. These observations suggest the course of CBF resembling the global ischemia followed by a continuous low-flow state in the early phase after SAH, which may lead to a persistent aerobic metabolism dysregulation in the brain [89]. Another study using the blood injection SAH model demonstrated that the extracellular accumulation of glutamate peaks at 1 day and lasts up to 7 days after SAH, which is accompanied by the persistent downregulation of glutamate and glutamine transporters in the brain, indicating a deranged glutamate-glutamine cycle after SAH [90]. Gerrit et al. also observed a similar pattern regarding the change of energy metabolism, such as glucose depletion as well as lactate and glutamate accumulation, which may result from acute hypoperfusion and cerebral autoregulation disruption after SAH. While a hypothermic intervention may mitigate such metabolic dysregulation after SAH [91]. All these studies suggest that CMD is a feasible way to detect early alternation in brain metabolism after SAH in real-time. More importantly, a major advantage of utilizing CMD in preclinical conditions is the feasibility to harvest post-CMD brain tissues, which is indeed inaccessible in live SAH patients, from SAH animals for a wide range of other biochemical or histological assays allowing for further characterization of the molecular mechanisms underlying the post-SAH brain metabolic dysregulation and its relation to the pathogenesis of EBI or long-term outcomes after SAH. One the other hand, experimental CMD study may enable us to simultaneously measure the brain metabolic changes in different brain regions [17] and thus provide us with a more comprehensive picture of post-SAH brain energy dysregulation, which is still lacking in the SAH research field. Thereby, more preclinical SAH studies involving the use of CMD and other methods are urgently needed. By analyzing the preclinical CMD data, we hope it could provide a bridge connecting back to clinical studies,

## 5. Conclusions

SAH is a debilitating condition that affects relatively young individuals with an extremely high mortality rate. It has been decades since the proposal of the EBI concept in SAH study; however, there is a huge barrier from the bench to the bedside, since most therapeutic approaches that successfully protect SAH animals against brain injury after SAH have failed in clinical trials [5]. Based on its limited invasiveness and feasibility, CMD may act as a bridge to or shorten or eliminate the gap between preclinical and human SAH studies [92]. However, optimization and standardization for the CMD procedure and data interpretation are still top priorities in SAH research. Besides, the feasibility of measuring additional biomarkers, such as large proteins and cytokines, by CMD in SAH remains to be further elucidated. Utilizing CMD, combined with other approaches, to measure disturbances in cerebral bioenergetic metabolism, as reflected by the altered concentration of energy metabolites in ISF and brain tissue, may allow us to characterize the temporal profile of cerebral metabolic dysfunction after SAH, which is essential to advance our understanding of the role of brain energy metabolism in post-SAH pathophysiology, and eventually, developing potential therapies that target the brain injury and thus to improve outcomes after SAH. 

## Figures and Tables

**Table 1:** Microdialysis findings from the last two years of clinical papers regarding subarachnoid hemorrhage and microdialysis.

Paper	Number of Patients	Patient Characteristics	Monitoring Period	Probe Location	Aim	Microdialysis Findings
[48]	51	Median age = 59 years Hunt & Hess grade 2 = 4 grade 3 = 12 grade 4 = 6 grade 5 = 29	Not specified	37% of patient’s normal brain tissue 63% patients perilesional.	Investigate the impact of catheter location on brain interstitial metabolite concentrations. Study temporal dynamics during monitoring time.	Glucopenia was independent of probe location. Probe placement in normal parenchyma may yield more relevant information than perilesional.
[49]	12	Mean age = 62 years GCS on admission 6 = 7 5 ≤ 5 Hunt & Hess grade 1–3 = 8 grade 4–5 = 4	Not specified	Right frontal cortex.	Investigate cerebral metabolic changes regarding CBF during therapeutic hypervolemia, hemodilution, & hypertension therapy in poor-grade SAH patients with DCI.	Global and regional CBF improved and the cerebral energy metabolic CMD parameters stayed statistically unchanged during HHH therapy in DCI patients.
[50]	190	Mean Age = 54 years Hunt & Hess grade 3 = 102 grade 4 = 54 grade 5 = 34	Not specified.	Ipsilateral frontal watershed area of the ruptured aneurysm	Assess the impact of invasive neuromonitoring on the detection rate of DCI and its influence on patient outcome.	Invasive neuromonitoring led to earlier detection of DCI events in poor-grade subarachnoid hemorrhage.
[51]	16	Mean age = 58 years WFNS All patients were grade 4 or 5	At the onset of subarachnoid hemorrhage for 4 days on average.	Bilaterally in MCA/ACA watershed territory	Assess the utility of a bilateral CMD in poor grade SAH patients to detect bilateral metabolic ischemic events to identify silent infarcts.	25% of ischemic events occurred in the contralateral hemisphere. The ipsilateral hemisphere showed no metabolic change
[52]	100	Mean Age = 58 years WFNS grade 1 = 5 grade 2 = 11 grade 3 = 5 grade 4 = 14 grade 5 = 63 GCS on admission = 3–7	11 days	White matter of the hemisphere deemed at greatest risk	Quantify the brain tissue hypoxia burden in subarachnoid hemorrhage and identify the simultaneous occurrence of pathologic values potentially amenable to treatment.	Elevated LPR and decreased glucose suggests anaerobic metabolism. Brain metabolic distress was linked to higher hospital mortality in severely brain injured patients.
[53]	36	Mean age = 56 years Hunt & Hess grade 3 = 4 grade 4 = 12 grade 5 = 20 Average GCS ≤ 8 on admission	Onset of subarachnoid hemorrhage for 7 days	Not specified	Investigate the relationship between neuroglobin and brain metabolism in subarachnoid hemorrhage patients.	Weak correlation between microdialysis metabolite concentrations and serum neuroglobin concentration.
[54]	10	Mean age = 51 years Hunt & Hess all patients were grade 3 or 4. Average GCS on admission = 3.5	1 h before procedure and 12 h after the procedure.	1 to 2 cm anterior to Kocher’s point into a watershed area ipsilateral to the ruptured aneurysm.	Investigate the effects of intra-arterial papaverine-hydrochloride on cerebral metabolism and oxygenation.	LPR decreased between 2–5 h after the procedure. Pyruvate remained elevated for 10 h. Lactate peaked at 1 h and 5–8 h.
[55]	30	Mean Age = 59 years Hunt & Hess grade 1 = 2 grade 2 = 6 grade 3 = 9 grade 4 = 11 grade 5 = 2 GCS on admission grade 1 = 1 grade 2 = 1 grade 4 = 6 grade 5 = 4 grade 6 = 18	3 days following onset of symptoms.	Right frontal lobe cortex.	Determine if low cerebral blood flow (CBF) measurements and pathological microdialysis parameters measured at the bedside can be observed early in patients with SAH who later developed DCI.	Low CBF, high lactate, and an increased L/P ratio were seen early after hemorrhage (Days 0–3) in patients who later developed DCI before any clinical signs had appeared.
[56]	17	Mean age = 57 years Hunt & Hess grade 2 = 2 grade 3 = 6 grade 4 = 2 grade 5 = 7	6 h from enteral food administration.	Frontal lobe white matter.	Investigate the effect of EN on brain metabolism in a cohort of poor-grade SAH patients	Enteral nutrition increases the concentration of glucose in cerebral ISF.
[57]	24	Median age = 60 years GCS of all patients was ≤ 8 on admission Hunt & Hess average = grade 4	Not specified	Hemisphere most at risk of ischemia and 10mm from lesion.	Investigate the effect of parenteral diclofenac infusion as routine fever treatment on brain extracellular IL-6 levels.	Shows that brain ISF IL-6 concentrations significantly decrease 2 h after diclofenac treatment.

## References

[1] Linn F.H., Rinkel G.J., Algra A., van Gijn J. (1996). Incidence of subarachnoid hemorrhage: Role of region, year, and rate of computed tomography: A meta-analysis. Stroke.

[2] van Gijn J., Kerr R.S., Rinkel G.J. (2007). Subarachnoid haemorrhage. Lancet.

[3] Hankey G.J., Jamrozik K., Broadhurst R. (2000). Epidemiology of aneurysmal subarachnoid hemorrhage in Australia and New Zealand: Incidence and case fatality from the Australasian Cooperative Research on Subarachnoid Hemorrhage Study (ACROSS). Stroke.

[4] Hop J.W., Rinkel G.J., Algra A., van Gijn J. (1997). Case-fatality rates and functional outcome after subarachnoid hemorrhage: A systematic review. Stroke.

[5] Sehba F.A., Hou J., Pluta R.M., Zhang J.H. (2012). The importance of early brain injury after subarachnoid hemorrhage. Prog. Neurobiol..

[6] Korja M., Lehto H., Juvela S., Kaprio J. (2016). Incidence of subarachnoid hemorrhage is decreasing together with decreasing smoking rates. Duodecim.

[7] Leclerc J.L., Garcia J.M., Diller M.A., Carpenter A.M., Kamat P.K., Hoh B.L., Dore S. (2018). A Comparison of Pathophysiology in Humans and Rodent Models of Subarachnoid Hemorrhage. Front. Mol. Neurosci..

[8] Rass V., Helbok R. (2019). Early Brain Injury After Poor-Grade Subarachnoid Hemorrhage. Curr. Neurol. Neurosci. Rep..

[9] Vagnozzi R., Signoretti S., Cristofori L., Alessandrini F., Floris R., Isgro E., Ria A., Marziali S., Zoccatelli G., Tavazzi B. (2010). Assessment of metabolic brain damage and recovery following mild traumatic brain injury: A multicentre, proton magnetic resonance spectroscopic study in concussed patients. Brain.

[10] Zahra K., Gopal N., Freeman W.D., Turnbull M.T. (2019). Using Cerebral Metabolites to Guide Precision Medicine for Subarachnoid Hemorrhage: Lactate and Pyruvate. Metabolites.

[11] Tholance Y., Barcelos G., Dailler F., Perret-Liaudet A., Renaud B. (2015). Clinical Neurochemistry of Subarachnoid Hemorrhage: Toward Predicting Individual Outcomes via Biomarkers of Brain Energy Metabolism. ACS Chem. Neurosci..

[12] Helbok R., Ko S.B., Schmidt J.M., Kurtz P., Fernandez L., Choi H.A., Connolly E.S., Lee K., Badjatia N., Mayer S.A. (2011). Global cerebral edema and brain metabolism after subarachnoid hemorrhage. Stroke.

[13] Hutchinson P.J., Jalloh I., Helmy A., Carpenter K.L., Rostami E., Bellander B.M., Boutelle M.G., Chen J.W., Claassen J., Dahyot-Fizelier C. (2015). Consensus statement from the 2014 International Microdialysis Forum. Intensive Care Med..

[14] Oddo M., Schmidt J.M., Carrera E., Badjatia N., Connolly E.S., Presciutti M., Ostapkovich N.D., Levine J.M., Le Roux P., Mayer S.A. (2008). Impact of tight glycemic control on cerebral glucose metabolism after severe brain injury: A microdialysis study. Crit. Care Med..

[15] Zeiler F.A., Thelin E.P., Helmy A., Czosnyka M., Hutchinson P.J.A., Menon D.K. (2017). A systematic review of cerebral microdialysis and outcomes in TBI: Relationships to patient functional outcome, neurophysiologic measures, and tissue outcome. Acta Neurochir. (Wien.).

[16] Dorsch N. (2011). A clinical review of cerebral vasospasm and delayed ischaemia following aneurysm rupture. Acta Neurochir. Suppl..

[17] Marzatico F., Gaetani P., Rodriguez y Baena R., Silvani V., Paoletti P., Benzi G. (1988). Bioenergetics of different brain areas after experimental subarachnoid hemorrhage in rats. Stroke.

[18] Bederson J.B., Germano I.M., Guarino L. (1995). Cortical blood flow and cerebral perfusion pressure in a new noncraniotomy model of subarachnoid hemorrhage in the rat. Stroke.

[19] Nornes H. (1973). The role of intracranial pressure in the arrest of hemorrhage in patients with ruptured intracranial aneurysm. J. Neurosurg..

[20] Tso M.K., Macdonald R.L. (2013). Acute microvascular changes after subarachnoid hemorrhage and transient global cerebral ischemia. Stroke Res. Treat..

[21] Oddo M., Levine J.M., Frangos S., Maloney-Wilensky E., Carrera E., Daniel R.T., Levivier M., Magistretti P.J., LeRoux P.D. (2012). Brain lactate metabolism in humans with subarachnoid hemorrhage. Stroke.

[22] Sehba F.A., Bederson J.B. (2006). Mechanisms of acute brain injury after subarachnoid hemorrhage. Neurol. Res..

[23] Bederson J.B., Levy A.L., Ding W.H., Kahn R., DiPerna C.A., Jenkins A.L., Vallabhajosyula P. (1998). Acute vasoconstriction after subarachnoid hemorrhage. Neurosurgery.

[24] Sarrafzadeh A., Haux D., Sakowitz O., Benndorf G., Herzog H., Kuechler I., Unterberg A. (2003). Acute focal neurological deficits in aneurysmal subarachnoid hemorrhage: Relation of clinical course, CT findings, and metabolite abnormalities monitored with bedside microdialysis. Stroke.

[25] Ankarcrona M., Dypbukt J.M., Bonfoco E., Zhivotovsky B., Orrenius S., Lipton S.A., Nicotera P. (1995). Glutamate-induced neuronal death: A succession of necrosis or apoptosis depending on mitochondrial function. Neuron.

[26] Keep R.F., Andjelkovic A.V., Stamatovic S.M., Shakui P., Ennis S.R. (2005). Ischemia-induced endothelial cell dysfunction. Acta Neurochir. Suppl..

[27] Helbok R., Kofler M., Schiefecker A.J., Gaasch M., Rass V., Pfausler B., Beer R., Schmutzhard E. (2017). Clinical Use of Cerebral Microdialysis in Patients with Aneurysmal Subarachnoid Hemorrhage-State of the Art. Front. Neurol..

[28] Sehba F.A., Schwartz A.Y., Chereshnev I., Bederson J.B. (2000). Acute decrease in cerebral nitric oxide levels after subarachnoid hemorrhage. J. Cereb. Blood Flow Metab..

[29] Geraghty J.R., Testai F.D. (2017). Delayed Cerebral Ischemia after Subarachnoid Hemorrhage: Beyond Vasospasm and Towards a Multifactorial Pathophysiology. Curr. Atheroscler. Rep..

[30] Etminan N., Vergouwen M.D., Ilodigwe D., Macdonald R.L. (2011). Effect of pharmaceutical treatment on vasospasm, delayed cerebral ischemia, and clinical outcome in patients with aneurysmal subarachnoid hemorrhage: A systematic review and meta-analysis. J. Cereb. Blood Flow Metab..

[31] Rowland M.J., Hadjipavlou G., Kelly M., Westbrook J., Pattinson K.T. (2012). Delayed cerebral ischaemia after subarachnoid haemorrhage: Looking beyond vasospasm. Br. J. Anaesth..

[32] Woitzik J., Dreier J.P., Hecht N., Fiss I., Sandow N., Major S., Winkler M., Dahlem Y.A., Manville J., Diepers M. (2012). Delayed cerebral ischemia and spreading depolarization in absence of angiographic vasospasm after subarachnoid hemorrhage. J. Cereb. Blood Flow Metab..

[33] Friedrich B., Muller F., Feiler S., Scholler K., Plesnila N. (2012). Experimental subarachnoid hemorrhage causes early and long-lasting microarterial constriction and microthrombosis: An in-vivo microscopy study. J. Cereb. Blood Flow Metab..

[34] Lucke-Wold B.P., Logsdon A.F., Manoranjan B., Turner R.C., McConnell E., Vates G.E., Huber J.D., Rosen C.L., Simard J.M. (2016). Aneurysmal Subarachnoid Hemorrhage and Neuroinflammation: A Comprehensive Review. Int. J. Mol. Sci..

[35] Pradilla G., Chaichana K.L., Hoang S., Huang J., Tamargo R.J. (2010). Inflammation and cerebral vasospasm after subarachnoid hemorrhage. Neurosurg. Clin. N. Am..

[36] Omerhodzic I., Dizdarevic K., Rotim K., Hajdarpasic E., Niksic M., Bejtic-Custovic E., Selimovic E., Custovic M. (2011). Cerebral microdialysis: Perioperative monitoring and treatment of severe neurosurgical patient. Acta Clin. Croat..

[37] Cesarini K.G., Enblad P., Ronne-Engstrom E., Marklund N., Salci K., Nilsson P., Hardemark H.G., Hillered L., Persson L. (2002). Early cerebral hyperglycolysis after subarachnoid haemorrhage correlates with favourable outcome. Acta Neurochir. (Wien.).

[38] Carteron L., Solari D., Patet C., Quintard H., Miroz J.P., Bloch J., Daniel R.T., Hirt L., Eckert P., Magistretti P.J. (2018). Hypertonic Lactate to Improve Cerebral Perfusion and Glucose Availability After Acute Brain Injury. Crit. Care Med..

[39] Zetterling M., Hillered L., Enblad P., Karlsson T., Ronne-Engström E. (2011). Relation between brain interstitial and systemic glucose concentrations after subarachnoid hemorrhage. J. Neurosurg..

[40] Tholance Y., Barcelos G.K., Dailler F., Renaud B., Marinesco S., Perret-Liaudet A. (2015). Biochemical neuromonitoring of poor-grade aneurysmal subarachnoid hemorrhage: Comparative analysis of metabolic events detected by cerebral microdialysis and by retrograde jugular vein catheterization. Neurol. Res..

[41] Vespa P., Bergsneider M., Hattori N., Wu H.M., Huang S.C., Martin N.A., Glenn T.C., McArthur D.L., Hovda D.A. (2005). Metabolic crisis without brain ischemia is common after traumatic brain injury: A combined microdialysis and positron emission tomography study. J. Cereb. Blood Flow Metab..

[42] Anderson C.M., Swanson R.A. (2000). Astrocyte glutamate transport: Review of properties, regulation, and physiological functions. Glia.

[43] van Dijk B.J., Vergouwen M.D., Kelfkens M.M., Rinkel G.J., Hol E.M. (2016). Glial cell response after aneurysmal subarachnoid hemorrhage—Functional consequences and clinical implications. Biochim. Biophys. Acta.

[44] Peerdeman S.M., Girbes A.R., Vandertop W.P. (2000). Cerebral microdialysis as a new tool for neurometabolic monitoring. Intensive Care Med..

[45] Bellander B.M., Cantais E., Enblad P., Hutchinson P., Nordstrom C.H., Robertson C., Sahuquillo J., Smith M., Stocchetti N., Ungerstedt U. (2004). Consensus meeting on microdialysis in neurointensive care. Intensive Care Med..

[46] Helbok R., Schiefecker A.J., Beer R., Dietmann A., Antunes A.P., Sohm F., Fischer M., Hackl W.O., Rhomberg P., Lackner P. (2015). Early brain injury after aneurysmal subarachnoid hemorrhage: A multimodal neuromonitoring study. Crit. Care.

[47] Suzuki H., Nishikawa H., Kawakita F. (2018). Matricellular proteins as possible biomarkers for early brain injury after aneurysmal subarachnoid hemorrhage. Neural. Regen. Res..

[48] Kofler M., Gaasch M., Rass V., Schiefecker A.J., Ianosi B., Lindner A., Beer R., Stover J.F., Rhomberg P., Pfausler B. (2020). The Importance of Probe Location for the Interpretation of Cerebral Microdialysis Data in Subarachnoid Hemorrhage Patients. Neurocrit. Care.

[49] Engquist H., Lewen A., Hillered L., Ronne-Engstrom E., Nilsson P., Enblad P., Rostami E. (2020). CBF changes and cerebral energy metabolism during hypervolemia, hemodilution, and hypertension therapy in patients with poor-grade subarachnoid hemorrhage. J. Neurosurg..

[50] Veldeman M., Albanna W., Weiss M., Conzen C., Schmidt T.P., Schulze-Steinen H., Wiesmann M., Clusmann H., Schubert G.A. (2020). Invasive neuromonitoring with an extended definition of delayed cerebral ischemia is associated with improved outcome after poor-grade subarachnoid hemorrhage. J. Neurosurg..

[51] Torne R., Culebras D., Sanchez-Etayo G., Garcia-Garcia S., Munoz G., Llull L., Amaro S., Heering C., Blasco J., Zavala E. (2020). Double hemispheric Microdialysis study in poor-grade SAH patients. Sci. Rep..

[52] Rass V., Solari D., Ianosi B., Gaasch M., Kofler M., Schiefecker A.J., Miroz J.P., Morelli P., Thome C., Beer R. (2019). Protocolized Brain Oxygen Optimization in Subarachnoid Hemorrhage. Neurocrit. Care.

[53] Ding C.Y., Kang D.Z., Wang Z.L., Lin Y.X., Jiang C.Z., Yu L.H., Wang D.L., Lin Z.Y., Gu J.J. (2019). Serum Ngb (Neuroglobin) Is Associated With Brain Metabolism and Functional Outcome of Aneurysmal Subarachnoid Hemorrhage. Stroke.

[54] Hosmann A., Wang W.T., Dodier P., Bavinzski G., Engel A., Herta J., Plochl W., Reinprecht A., Gruber A. (2020). The Impact of Intra-Arterial Papaverine-Hydrochloride on Cerebral Metabolism and Oxygenation for Treatment of Delayed-Onset Post-Subarachnoid Hemorrhage Vasospasm. Neurosurgery.

[55] Rostami E., Engquist H., Howells T., Johnson U., Ronne-Engstrom E., Nilsson P., Hillered L., Lewen A., Enblad P. (2018). Early low cerebral blood flow and high cerebral lactate: Prediction of delayed cerebral ischemia in subarachnoid hemorrhage. J. Neurosurg..

[56] Kofler M., Schiefecker A.J., Beer R., Gaasch M., Rhomberg P., Stover J., Pfausler B., Thome C., Schmutzhard E., Helbok R. (2018). Enteral nutrition increases interstitial brain glucose levels in poor-grade subarachnoid hemorrhage patients. J. Cereb. Blood Flow Metab..

[57] Schiefecker A.J., Rass V., Gaasch M., Kofler M., Thome C., Humpel C., Ianosi B., Hackl W.O., Beer R., Pfausler B. (2019). Brain Extracellular Interleukin-6 Levels Decrease Following Antipyretic Therapy with Diclofenac in Patients with Spontaneous Subarachnoid Hemorrhage. Hypothermia Temp. Manag..

[58] Peerdeman S.M., van Tulder M.W., Vandertop W.P. (2003). Cerebral microdialysis as a monitoring method in subarachnoid hemorrhage patients, and correlation with clinical events--a systematic review. J. Neurol..

[59] Skjøth-Rasmussen J., Schulz M., Kristensen S.R., Bjerre P. (2004). Delayed neurological deficits detected by an ischemic pattern in the extracellular cerebral metabolites in patients with aneurysmal subarachnoid hemorrhage. J. Neurosurg..

[60] Sarrafzadeh A.S., Sakowitz O.W., Kiening K.L., Benndorf G., Lanksch W.R., Unterberg A.W. (2002). Bedside microdialysis: A tool to monitor cerebral metabolism in subarachnoid hemorrhage patients?. Crit. Care Med..

[61] Chou S.H., Macdonald R.L., Keller E. (2019). Biospecimens and Molecular and Cellular Biomarkers in Aneurysmal Subarachnoid Hemorrhage Studies: Common Data Elements and Standard Reporting Recommendations. Neurocrit. Care.

[62] Bosche B., Graf R., Ernestus R.I., Dohmen C., Reithmeier T., Brinker G., Strong A.J., Dreier J.P., Woitzik J. (2010). Recurrent spreading depolarizations after subarachnoid hemorrhage decreases oxygen availability in human cerebral cortex. Ann. Neurol..

[63] Sakowitz O.W., Santos E., Nagel A., Krajewski K.L., Hertle D.N., Vajkoczy P., Dreier J.P., Unterberg A.W., Sarrafzadeh A.S. (2013). Clusters of spreading depolarizations are associated with disturbed cerebral metabolism in patients with aneurysmal subarachnoid hemorrhage. Stroke.

[64] Devanney N.A., Stewart A.N., Gensel J.C. (2020). Microglia and macrophage metabolism in CNS injury and disease: The role of immunometabolism in neurodegeneration and neurotrauma. Exp. Neurol..

[65] Hanafy K.A., Grobelny B., Fernandez L., Kurtz P., Connolly E.S., Mayer S.A., Schindler C., Badjatia N. (2010). Brain interstitial fluid TNF-alpha after subarachnoid hemorrhage. J. Neurol. Sci..

[66] Hanafy K.A., Stuart R.M., Khandji A.G., Connolly E.S., Badjatia N., Mayer S.A., Schindler C. (2010). Relationship between brain interstitial fluid tumor necrosis factor-alpha and cerebral vasospasm after aneurysmal subarachnoid hemorrhage. J. Clin. Neurosci..

[67] Sarrafzadeh A., Schlenk F., Gericke C., Vajkoczy P. (2010). Relevance of cerebral interleukin-6 after aneurysmal subarachnoid hemorrhage. Neurocritical Care.

[68] Graetz D., Nagel A., Schlenk F., Sakowitz O., Vajkoczy P., Sarrafzadeh A. (2010). High ICP as trigger of proinflammatory IL-6 cytokine activation in aneurysmal subarachnoid hemorrhage. Neurol. Res..

[69] Sarrafzadeh A., Copin J.C., Bengualid D.J., Turck N., Vajkoczy P., Bijlenga P., Schaller K., Gasche Y. (2012). Matrix metalloproteinase-9 concentration in the cerebral extracellular fluid of patients during the acute phase of aneurysmal subarachnoid hemorrhage. Neurol. Res..

[70] Schiefecker A.J., Dietmann A., Beer R., Pfausler B., Lackner P., Kofler M., Fischer M., Broessner G., Sohm F., Mulino M. (2017). Neuroinflammation is Associated with Brain Extracellular TAU-Protein Release After Spontaneous Subarachnoid Hemorrhage. Curr. Drug Targets.

[71] Stuart R.M., Schmidt M., Kurtz P., Waziri A., Helbok R., Mayer S.A., Lee K., Badjatia N., Hirsch L.J., Connolly E.S. (2010). Intracranial multimodal monitoring for acute brain injury: A single institution review of current practices. Neurocritical Care.

[72] Chen J.W., Rogers S.L., Gombart Z.J., Adler D.E., Cecil S. (2012). Implementation of cerebral microdialysis at a community-based hospital: A 5-year retrospective analysis. Surg. Neurol. Int..

[73] Chefer V.I., Thompson A.C., Zapata A., Shippenberg T.S. (2009). Overview of brain microdialysis. Curr. Protoc. Neurosci..

[74] Zetterstrom T., Sharp T., Collin A.K., Ungerstedt U. (1988). In vivo measurement of extracellular dopamine and DOPAC in rat striatum after various dopamine-releasing drugs; implications for the origin of extracellular DOPAC. Eur. J. Pharmacol..

[75] Zhou T., Kalanuria A. (2018). Cerebral Microdialysis in Neurocritical Care. Curr. Neurol. Neurosci. Rep..

[76] Bouras T.I., Gatzonis S.S., Georgakoulias N., Karatza M., Sianouni A., Stranjalis G., Boviatsis E., Vasileiou S., Sakas D.E. (2011). Neuro-inflammatory sequelae of minimal trauma in the non-traumatized human brain. A microdialysis study. J. Neurotrauma.

[77] Di Chiara G., Tanda G., Carboni E. (1996). Estimation of in-vivo neurotransmitter release by brain microdialysis: The issue of validity. Behav. Pharm..

[78] Bhatia R., Hashemi P., Razzaq A., Parkin M.C., Hopwood S.E., Boutelle M.G., Strong A.J. (2006). Application of rapid-sampling, online microdialysis to the monitoring of brain metabolism during aneurysm surgery. Neurosurgery.

[79] Carpenter K.L., Jalloh I., Gallagher C.N., Grice P., Howe D.J., Mason A., Timofeev I., Helmy A., Murphy M.P., Menon D.K. (2014). (13)C-labelled microdialysis studies of cerebral metabolism in TBI patients. Eur. J. Pharm. Sci..

[80] Wyss M.T., Magistretti P.J., Buck A., Weber B. (2011). Labeled acetate as a marker of astrocytic metabolism. J. Cereb. Blood Flow Metab..

[81] Shannon R.J., Carpenter K.L., Guilfoyle M.R., Helmy A., Hutchinson P.J. (2013). Cerebral microdialysis in clinical studies of drugs: Pharmacokinetic applications. J. Pharm. Pharm..

[82] Darvesh A.S., Carroll R.T., Geldenhuys W.J., Gudelsky G.A., Klein J., Meshul C.K., Van der Schyf C.J. (2011). In vivo brain microdialysis: Advances in neuropsychopharmacology and drug discovery. Expert Opin. Drug Discov..

[83] Kumar G., Shahripour R.B., Harrigan M.R. (2016). Vasospasm on transcranial Doppler is predictive of delayed cerebral ischemia in aneurysmal subarachnoid hemorrhage: A systematic review and meta-analysis. J. Neurosurg..

[84] de Oliveira Manoel A.L., Mansur A., Murphy A., Turkel-Parrella D., Macdonald M., Macdonald R.L., Montanera W., Marotta T.R., Bharatha A., Effendi K. (2014). Aneurysmal subarachnoid haemorrhage from a neuroimaging perspective. Crit. Care.

[85] Cremers C.H., van der Schaaf I.C., Wensink E., Greving J.P., Rinkel G.J., Velthuis B.K., Vergouwen M.D. (2014). CT perfusion and delayed cerebral ischemia in aneurysmal subarachnoid hemorrhage: A systematic review and meta-analysis. J. Cereb. Blood Flow Metab..

[86] Wagner M., Jurcoane A., Hildebrand C., Guresir E., Vatter H., Zanella F.E., Berkefeld J., Pilatus U., Hattingen E. (2013). Metabolic changes in patients with aneurysmal subarachnoid hemorrhage apart from perfusion deficits: Neuronal mitochondrial injury?. Am. J. Neuroradiol..

[87] Lama S., Auer R.N., Tyson R., Gallagher C.N., Tomanek B., Sutherland G.R. (2014). Lactate storm marks cerebral metabolism following brain trauma. J. Biol. Chem..

[88] Marbacher S., Gruter B., Schopf S., Croci D., Nevzati E., D’Alonzo D., Lattmann J., Roth T., Bircher B., Wolfert C. (2019). Systematic Review of In Vivo Animal Models of Subarachnoid Hemorrhage: Species, Standard Parameters, and Outcomes. Transl. Stroke Res..

[89] Westermaier T., Jauss A., Eriskat J., Kunze E., Roosen K. (2011). The temporal profile of cerebral blood flow and tissue metabolites indicates sustained metabolic depression after experimental subarachnoid hemorrhage in rats. Neurosurgery.

[90] Wu C.T., Wen L.L., Wong C.S., Tsai S.Y., Chan S.M., Yeh C.C., Borel C.O., Cherng C.H. (2011). Temporal changes in glutamate, glutamate transporters, basilar arteries wall thickness, and neuronal variability in an experimental rat model of subarachnoid hemorrhage. Anesth. Analg..

[91] Schubert G.A., Poli S., Mendelowitsch A., Schilling L., Thome C. (2008). Hypothermia reduces early hypoperfusion and metabolic alterations during the acute phase of massive subarachnoid hemorrhage: A laser-Doppler-flowmetry and microdialysis study in rats. J. Neurotrauma.

[92] Bourne J.A. (2003). Intracerebral microdialysis: 30 years as a tool for the neuroscientist. Clin. Exp. Pharm. Physiol.

